# From muscle to brain: the mediating effect of vitamin D in the relationship between muscle loss and cognitive function in Alzheimer’s disease

**DOI:** 10.3389/fnagi.2026.1630798

**Published:** 2026-05-13

**Authors:** Xiaoting Ma, Linlin Yao, Fangbo Li, Chong Cui, Erlei Wang, Caishan Wang, Ping Zhao, Xiaofen Weng, Chunfeng Liu, Shanwen Liu, Hua Hu

**Affiliations:** 1 Department of Neurology, The Second Affiliated Hospital of Soochow University, Suzhou, China; 2 Department of Imaging, The Second Affiliated Hospital of Soochow University, Suzhou, China; 3 Department of Ultrasound Imaging, The Second Affiliated Hospital of Soochow University, Suzhou, China; 4 Department of Geriatric Medicine, The Affiliated Suzhou Hospital of Nanjing Medical University, Suzhou Municipal Hospital, Suzhou, China

**Keywords:** Alzheimer’s disease, cognitive function, mediating effect, muscle loss, vitamin D

## Abstract

**Objective:**

To investigate the association among Vitamin D (VD), muscle loss and cognitive function, and further analyze the possible role of VD in the association between muscle loss and cognitive function in patients with Alzheimer’s disease (AD).

**Methods:**

This cross-sectional study included 58 patients with AD (30 mild and 28 moderate) and 30 neurotypical controls (NC). Demographic data, neuropsychological tests, and serum VD levels were collected. Muscle loss related indicators, including both clinical macroscopic and morphological microstructural indicators, were measured.

**Results:**

The differences among 3 groups in different cognitive domains, muscle loss related indicators and serum VD levels were statistically significant (*P* < 0.05). Adjusted for age, sex and BMI, correlations were observed among broad cognitive domains, muscle loss related indicators (especially muscle mass and upper limb muscle strength) and serum VD levels in patients with AD (*P* < 0.05). Mediation analyses revealed that muscle loss, particularly muscle mass reduction, significantly impacted cognitive function in patients with AD (*P* < 0.05). This effect remained statistically significant after accounting for VD levels (*P* < 0.05), and VD deficiency partially mediated the effect of muscle loss on cognitive function (*P* < 0.05).

**Conclusion:**

VD level, muscle loss, and cognitive dysfunction are closely related, and VD partially mediated the association between muscle mass reduction and cognitive dysfunction in patients with AD. These preliminary findings highlight the importance of assessing muscle loss and VD deficiency, and suggest that targeted intervention may be a valuable management strategy for the AD population.

## 1 Introduction

Alzheimer’s disease (AD) is a neurodegenerative disease with an insidious onset, characterized by progressive cognitive decline, accompanied by psychiatric and behavioral symptoms and varying degrees of impairment in daily functioning. It severely impacts the patients’ mental and physical health and quality of life, placing a heavy psychological and economic burden on families and society. The global AD population has exceeded 40 million, with the prevalence doubling every 5 years in people over 65 years of age and affecting nearly 50% of individuals aged 85 and older (Nichols et al., 2019). Given the increase in aging population and the irreversibly progression of AD, early detection, accurate diagnosis, and effective intervention for AD are crucial.

Muscle loss, particularly in the appendicular skeletal muscle, refers to an age-related muscle atrophy and decline in muscle function, involving loss of muscle mass, muscle strength and/or physical performance (Chen et al., 2024). It increases the risk of adverse health outcomes such as falls, mobility disability, and all-cause mortality (Kim et al., 2024). A recent meta-analysis revealed that the prevalence of muscle loss ranges from 10 to 27% in adults over 60 and increases exponentially with age (Amini et al., 2024). Actually, muscle loss onset commences in the fourth decade of life, progressing to 30–50% declines in muscle mass and functional capacity by the ninth decade (McCormick and Vasilaki, 2018). In recent years, it has been found that muscle loss is not only associated with aging, but is also independently linked to the risk of AD (Peng et al., 2020). For instance, a cohort study by Beeri et al. found that muscle loss was a risk factor for AD and mild cognitive impairment (MCI) in later life, and that severe muscle loss at baseline was linked to a higher prevalence of MCI and AD, as well as faster cognitive decline in later life (Beeri et al., 2021). A recent report from the annual meeting of the Radiological Society of North America (RSNA) shows that muscle loss, measured through temporalis muscle size via brain magnetic resonance imaging (MRI), is a risk factor for developing AD. The study found that older adults with smaller temporalis muscle size were 60% more likely to develop AD and experienced greater cognitive and brain volume decline over nearly 6 years (Moradi et al., 2024). A meta-analysis of 77 studies found that muscle loss increased the prevalence of MCI, AD and non-AD dementia by 58, 197, and 68%, respectively (Amini et al., 2024). All these studies suggest that muscle loss plays an important role in the development of AD. The “muscle-brain” axis hypothesis proposes that skeletal muscle participates in this process through the muscle-brain crosstalk network, the specific mechanisms include chronic low-grade inflammation (inflammaging), mitochondrial dysfunction, oxidative stress, insulin resistance, age-related hormonal changes, reduced secretion of myokines, physical inactivity, and malnutrition (Amini et al., 2024). However, it remains unclear whether there are clinically visualized mediators involved in the interaction between AD and muscle loss. Defined as variables that explain the mechanism through which an independent variable affects a dependent variable, these mediators are crucial for the formulation and development of targeted intervention strategies.

Vitamin D (VitD, VD), a lipophilic secosteroid hormone, mainly includes vitamin D_2_ and vitamin D_3_. In addition to dietary intake, VD can be synthesized in the skin from 7-dehydrocholesterol via ultraviolet light, and then hydroxylated to form 25-hydroxyvitamin D (25OHD) in the liver by enzymes such as CYP2R1. Subsequently, 25OHD is further converted to 1,25-dihydroxyvitamin D [1,25(OH)2D], which is the active form of VD, in the kidney or extra-renal tissues by enzymes such as CYP27B1. This hormonal metabolite acts through nuclear VD receptor (VDR)-mediated genomic regulation, to modulate multiple organ functions, including skeletal muscle and the brain, and is implicated in the regulation of various diseases. Epidemiological data indicate that the global prevalence of vitamin D deficiency (VDD) is estimated to be between 60 and 80%, and it is even higher in older adults (van Schoor and Lips, 2017). Concentrations of 7-dehydrocholesterol in aging skin decrease by about 50%, VD production by aging skin decreases shows a reduction of about 40%, with an estimated decline of 13% per decade (She et al., 2021). Current research suggests that VD is strongly associated with age-related muscle loss and cognitive dysfunction. For instance, a Korean cohort study revealed that low VD levels were associated with reduced muscle mass, reduced grip strength, and limited physical activity (Lee et al., 2023). A Canadian intervention trial, with a 10-year follow-up, found that adequate VD supplementation was associated with a 40% reduction in the incidence of AD in healthy individuals, and a significant reduction in the incidence of AD after VD supplementation in individuals with cognitive dysfunction at baseline (Ghahremani et al., 2023). A team of researchers in Shanghai, China, found 46.8% lower incident cognitive impairment with VD supplementation (OR = 0.53) in healthy elders, and 60.5% reduced AD conversion risk in MCI patients receiving 12-month intervention of VD supplementation (Jiang et al., 2023). Sarah L. Booth’s team recently provided the first quantification of VD in brain regions (including middle temporal cortex, middle frontal cortex, cerebellum, and white matter of the anterior watershed brain) via the Rush Memory and Aging Projec**t** (MAP). They found that brain VD levels were inversely associated with AD and MCI risk. Notably, their results also showed that circulating blood VD levels reflect brain VD concentrations (Shea et al., 2023). It is noteworthy that VDR and key enzymes for VD synthesis and metabolism, such as CYP27B1, are highly expressed in both skeletal muscle and the brain (the hippocampus, etc.). Furthermore, VD has been confirmed to participate in diverse molecular signaling pathways in both skeletal muscle and the brain (Annweiler et al., 2015; Caballero-García et al., 2021; Pál et al., 2023). Collectively, these findings suggest that VD may serve as an important link within the “muscle-brain” axis. Despite current evidence supporting that VD level is closely related to muscle loss and AD, most of the related studies are limited to bivariate correlations, and there is a lack of research on the interaction of VD between AD and muscle loss. Therefore, based on the “muscle-brain” axis hypothesis (Burtscher et al., 2021), this study aims to apply more accurate indicators to evaluate muscle structure and function, and cognitive function in patients with AD. A multidimensional assessment framework will be developed to evaluate VD levels, muscle loss, and cognitive function, further analyze their interrelations, and explore the possible role of VD in the relationship between muscle loss and cognitive function in AD. This will provide a theoretical basis for early detection, targeted intervention, and comprehensive diagnosis and treatment of patients with AD.

## 2 Materials and methods

### 2.1 Participants

This cross-sectional study included 58 patients with mild to moderate AD diagnosed in the memory disorder clinic (30 mild and 28 moderate), as well as 30 matched neurotypical controls (NC) recommended by the Physical Examination Center from the Department of Neurology in the Second Affiliated Hospital of Soochow University from October 2023 to March 2025. Participants were consecutive recruited from the outpatient department during the study period.

Inclusion criteria for AD group: ➀Meeting the 2011 National Institute on Aging and Alzheimer’s Association (NIA-AA) core diagnostic criteria for probable AD dementia (McKhann et al., 2011) and the presence of amyloid deposits confirmed by Positron emission tomography (PET) (Jack et al., 2018). ➁Age between 60 and 85 years, right-handed. ➂Mini-Mental State Examination (MMSE) score: illiterate ≤ 22, primary school ≤ 23, secondary school ≤ 24, and university ≤ 26. According to MMSE, the severity of AD was further classified into mild (21 ≤ MMSE ≤ 26) and moderate (11 ≤ MMSE ≤ 20) (Shigemori et al., 2010). ➃3.0T (Siemens, Germany, Prisma) MRI showed no space-occupying lesions and age-inappropriate periventricular and deep white matter lesions (Fazekas score ≤ 2). ➄Hachinski ischemia score ≤ 4, to exclude significant vascular contributions to dementia. ➅Participants had not taken any medications known to impact cognitive function or mental status, nor any vitamin D supplements, within 1 month before enrollment. ➆Neuropsychiatric Inventory (NPI) score < 4 points, to minimize the confounding influence of significant neuropsychiatric symptoms on cognitive assessment. Exclusion criteria: ➀Other diseases that can cause cognitive impairment, such as cerebrovascular disease (CVA, including history of stroke or relevant infarcts/hemorrhages on the MRI obtained per inclusion criterion ➃), brain tumor (identified via history or on the same MRI). ➁Serious medical diseases, such as chronic cardiopulmonary, hepatic and renal insufficiency. ➂Severe depression and other mental diseases. ➃Incomplete clinical data, unable to cooperate with the cognitive function examination. ➄Those who are unable to perform Bioelectric Impedance Analysis (BIA) and MRI due to the placement of metal or cardiac pacemaker in the body. ➅Diseases affecting limb movements such as lumbar disc herniation, history of bone fracture, osteoarthrosis, etc. ➆Combined with symptoms of the motor system, such as muscle weakness, myasthenia gravis, and signs of cone system and extrapyramidal system damage on examination.

Inclusion criteria for NC group: ➀Age between 60 and 85 years, right-handed. ➁Independent behavioral ability without cognitive dysfunction or manifested as memory loss. ➂MMSE ≥ 27 points (Shigemori et al., 2010). ➃MRI performed confirming the white matter of the brain is normal in the imaging examination, and there is no central nervous system disease. ➄Participants had not taken any medications known to impact cognitive function or mental status, nor any vitamin D supplements, within 1 month before enrollment. The exclusion criteria were the same as the AD group.

The study was approved by the Ethics Committee of the hospital (Approval No. JD-LK-2021-049-01), and subjects and family members of patients with AD signed an informed consent.

### 2.2 General information

Age, sex, education, AD course, medical history, personal history, hearing, height, weight, body mass index (BMI), activities of daily living (ADL), mini nutritional assessment (MNA), dietary diversity score (DDS) were collected. Medical history included hypertension, diabetes mellitus, hyperlipidemia, coronary heart disease (CHD) and other conditions. Personal history included history of smoking and alcohol consumption. BMI is calculated as weight (kg)/height (m)^2^.

### 2.3 Neuropsychological assessment

The evaluators underwent professional standardization training and conducted neuropsychological tests in a quiet room. Non-cognitive assessments included emotional and sleep related tests: HAMD, Hamilton depression scale; Hamilton anxiety scale (HAMA); Pittsburgh sleep quality index (PSQI); Athens insomnia scale (AIS); Epworth sleepiness scale (ESS). Cognitive function assessments included: the Chinese version of the MMSE, the Beijing version of the Montreal cognitive assessment (MoCA), the memory and executive screening scale (MES), the clock drawing test (CDT), animal fluency task (AFT), Boston naming test (BNT), digital span test (DST), digit symbol substitution test (DSST), and clinical dementia rating (CDR). The Chinese version of the MMSE and the Beijing version of the MoCA assess comprehensive cognitive functioning. CDT assesses executive and visuospatial abilities. AFT assesses executive and language abilities. BNT assesses naming abilities in the language domain. The total score of the MES (MES-T) includes the Memory Factor Score (MES-M), which assesses episodic memory, and the Executive Factor Score (MES-E) to assess executive function. DST contains both forward (FDST) and backward (BDST) components to assess attention, with the BDST also assessing working memory for executive function. DSST assesses speed of information processing, which considered kind of executive function. CDR assesses severity of dementia by using the CDR-global score (CDR-GS) (Supplementary Table S1).

### 2.4 Muscle loss related clinical macroscopic indicators measured by comprehensive clinical assessment tool

Clinical macroscopic indicators related to muscle loss include: ➀appendicular skeletal muscle mass index (ASMI): This evaluates muscle mass. Applying BIA (DBA-610), subject’s feet are placed on foot electrodes, and the hands grip the handles to ensure the thumbs fully contact the electrodes with arms are naturally abducted 30°, remaining still during the measurement. ASMI calculated as appendicular muscle mass (kg)/height^2^ (m^2^). Cutoffs: male ≤ 7.0 kg/m^2^, female ≤ 5.7 kg/m^2^. ➁Grip strength: This assesses upper limb muscle strength. Grip strength was measured with a spring-loaded dynamometer, with the subject sitting upright, arms naturally bent at a 90-degree angle, and using the dominant hand to grip the device with maximal force. At least three trials are conducted, and the highest reading is selected. Cutoffs: male < 28.0 kg, female < 18.0 kg. ➂Chair stand test: This assesses lower limb muscle strength and physical performance. Using a 46 cm high, armless chair, the subject crossed his or her arms in front the chest and completed 5 consecutive sit-to-stand movements as quickly as possible without using arms, and the time taken was recorded. Cutoffs: ≥ 12 s. ➃Gait speed: This main assessment of physical performance: A stopwatch is used to measure the time taken to walk 6 m at a normal pace. Cutoffs: < 1.0 m/s. ➄Short physical performance battery (SPPB): This includes chair stand test and gait speed mentioned above, and balance tests. Cutoffs: ≤ 9 score. Cut-off values were adopted from the Asian Working Group for Sarcopenia (AWGS) 2019 consensus for the diagnosis of sarcopenia (Chen et al., 2020).

### 2.5 Morphological microstructural indicators assessed by multimodal muscle ultrasonography

A Supersonic AixPlorer (Lucco, France) ultrasound diagnostic instrument, SL15-4 high-frequency linear array probe, with a frequency of 4–15 MHz, was used to perform ultrasound examinations of the appendicular skeletal muscles by two board-certified physicians with over 10 years of experience specializing in musculoskeletal ultrasound. blinded to the clinical diagnosis of the participants. The musculoskeletal parameters of the skeletal muscles of the extremities included: ➀muscle thickness (MT): the distance between the superficial and deep muscle fascia; ➁Cross-sectional area (CSA): the area of the muscle perpendicular to its fibers; ➂Fascicle length (FL): the length of the muscle fibers between the superficial and deep fascia; ➃Pennation angle (PA): the angle at which the muscle fibers are inserted into the deep fascia. ➄Young’s modulus (YM). Shear wave elastography (SWE) was employed to quantitatively measure muscle stiffness by obtaining YM, which reflects the shear wave propagation speed. Within the elastic limit of the tissue, the higher the stiffness, the greater the value of YM and the faster the shear wave velocity. Considering the inconvenience in exposing the patient’s thigh and the substantial impact of widespread degenerative knee joint changes on the thigh muscles (Isaka et al., 2019), representative appendicular muscles on the dominant side of the subject were selected, including the biceps brachii from the upper arm, the brachioradialis from the forearm, and the gastrocnemius from the calf muscle (Supplementary Section 2). Given the brief duration of each muscle contraction, the subject is asked to perform three contractions, with a 20s rest period between each. The ultrasound technician will conduct three measurements to capture elasticity imaging and corresponding average values in the contracted state (each image containing two measurements), and the final average value will be computed (derived from six average values). Furthermore, the measurement times for FL, PA, and YM in the contracted state are kept to ≤ 10 s to prevent measurement errors caused by muscle fatigue.

### 2.6 Measurement of serum VD levels

Participants were examined at the hospital in the morning after waking up after fasting for about 12 h. At about 8 a.m., 5 mL of blood was collected from the elbow vein and placed in sterile tubes for analysis. Mass spectrometry was employed using the Waters Acquity UPLC system (Waters Corporation, United States) and the API 4000 Qtrap tandem quadrupole mass spectrometer (AB SCIEX, United States) to measure 25OHD_2_, 25OHD_3_, and total 25OHD concentration in serum.

25OHD is the main form of circulating VD, and serum 25OHD concentration is recommended to reflect the VD status in the body. According to the degree of deficiency of VD, this study divided the participants into VD deficiency group (VDD) and non-deficiency VD group (VDnD) based on whether their 25OHD level was greater than 20 ng/mL (Lee et al., 2023).

### 2.7 Statistical analysis

Statistical Package for the Social Sciences (SPSS, version 26.0, IBM Corp.) software was used for data analysis. Normally distributed continuous variables were expressed as $\bar{x}$ ± s and one-way Analysis of variance (ANOVA) was applied for comparisons between the three groups, with pairwise comparisons performed using the Least Significant Difference (LSD) test. Non-normally distributed continuous variables were expressed as M (Q1, Q3). Kruskal-Wallis H test was used for group comparisons, and pairwise comparisons were performed using the Bonferroni test for *post hoc* analysis. Categorical variables were presented as frequency (percentage), and the χ^2^-test was used for group comparisons. Partial correlation analysis was applied to assess the relationships between cognitive function, muscle loss, and VD level. Independent and dependent variables were selected based on prior literature and statistical relevance. Covariate selection was based on variables widely found to be related to cognitive function, muscle loss, and VD level in the literature, such as sex, age, and BMI. Multiple linear regression equations were used to construct three models and to examine the mediating effects of the VD level in the relationship between muscle loss and cognitive function in patients with AD. A bootstrap sampling test (5,000 iterations) was employed for mediation analysis to estimate path effects, using a 95% bias-corrected confidence interval (CI). In the mediation analysis, continuous variables were standardized. The significance level was set at α = 0.05. The minimum sample size was determined through an *a priori* power analysis via G*Power 3.1.9.7, while also considering the practical feasibility of recruiting specific clinical cohorts. For the intra-group correlation analysis within the AD group (*n* = 58), a sample of this size provides a statistical power of approximately 0.78 to detect a medium effect size of | *r*| = 0.35 (α = 0.05). The total cohort (*n* = 88) provides a power of 0.80 to detect an effect size of *f*^2^ = 0.15 in the mediation and regression models.

## 3 Results

### 3.1 Comparison of general information and clinical characteristics between AD and NC groups

This study included 30 NC and 58 patients with AD (28 mild and 30 moderate). There were no significant differences in general demographic data among 3 groups. Comparing the nutritional indicators, the moderate AD group had lower BMI than the NC group (*P* < 0.05). Comparing the neuropsychological scale scores among 3 groups, there were significant differences in MMSE, MoCA, MES-E, MES-T, AFT, BNT, BDST, FDST, DST, and DSST scores, in addition, the moderate AD group had lower CDT scores compared to both the NC and mild AD group (all *P* < 0.05).

The comparison of the muscle loss related indicators among three groups showed that, regarding clinical macroscopic indicators, the moderate AD group had lower ASMI, grip strength, and longer Chair stand test time than the NC group (all *P* < 0.05). In terms of morphological microstructure indicators, there were significant differences in MT, CSA and MT/CSA of brachialis, PA of gastrocnemius among 3 groups (all *P* < 0.05). In addition, the mild and moderate AD groups had lower MT, CSA and MT/CSA of biceps brachii and gastrocnemius, as well as PA-T than the NC group (all *P* < 0.05). The moderate AD group had lower PA-C than both the NC and mild AD group, and the moderate AD group had lower YM of biceps brachii, YM-C, YM-T, △YM, and LF/MT of gastrocnemius than the NC group (all *P* < 0.05).

In the comparison of the serum VD levels, there were significant differences among three groups in 25OHD and 25OHD_3_(all *P* < 0.05). The number of VDD cases were 2 cases (6.7%) in the NC group, 10 cases (35.7%) in the mild AD group, and 23 cases (76.7%) in the moderate AD group (Table 1).

**TABLE 1:** Comparison of general information and clinical characteristics between AD and control groups.

Variable	NC (*n* = 30)	Mild AD(*n* = 28)	Moderate AD(*n* = 30)	*F/H/*χ ^2^ value	*P-*value
Demographic data					
Age (years, x̄ ± s)	73.67 ± 7.20	74.25 ± 5.27	74.43 ± 6.84	0.113^a^	0.894
Sex [n(%)]				4.287^a^	0.117
Male	18(60.0)	13(46.4)	10(33.3)		
Female	12(40.0)	15(53.6)	20(66.7)		
Education (years, x̄ ± s)	6.43 ± 4.52	7.34 ± 3.64	6.60 ± 4.81	0.350^a^	0.706
Course [years, M(Q1, Q3)]	0.0(0.0, 0.0)	2.0(1.0, 2.9) ^†^	2.0(1.0, 3.3) ^‡^	62.041^b^	<0.001
Hypertension [n(%)]	13(43.3)	18(64.3)	17(56.7)	2.647^c^	0.266
Diabetes mellitus [n(%)]	9(30.0)	6(21.4)	9(30.0)	0.707^c^	0.702
Hyperlipidemia [n(%)]	10(33.3)	12(42.9)	10(33.3)	0.748^c^	0.688
CHD [n(%)]	1(3.3)	1(3.6)	5(16.7)	4.437^c^	0.109
Alcohol drinking [n(%)]	6(20.0)	4(14.3)	4(13.3)	0.565^c^	0.754
Current smoking [n(%)]	2(6.7)	2(7.1)	4(13.3)	0.950^c^	0.622
Hearing [n(%)]	6(20.0)	6(21.4)	5(16.7)	0.224^c^	0.894
Nutritional status indicators					
BMI [kg/m^2^, M(Q1, Q3)]	24.0(22.5, 25.0)	22.6(20.8, 24.1)	21.1(19.4, 23.6) ^‡^	10.370^b^	0.006
MNA [score, M(Q1, Q3)]	26.5(25.0, 28.0)	26.3(25.7, 28.4)	25.3(23.0, 27.1)	5.878^b^	0.053
DDS [score, M(Q1, Q3)]	7.0(6.0, 8.3)	7.0(6.0, 8.0)	6.0(5.8, 7.3) ^‡^	5.148^b^	0.076
Neuropsychological scale scores-non-cognitive assessment					
HAMD [score, M(Q1, Q3)]	3.5(1.8, 7.0)	2.0(0.0, 4.8)	3.0(2.0, 7.5)	3.752^b^	0.153
HAMA [score, M(Q1, Q3)]	2.0(0.8, 4.3)	2.0(1.0, 6.0)	3.0(2.0, 5.3)	3.552^b^	0.169
PSQI [score, M(Q1, Q3)]	4.0(2.0, 6.3)	4.0(2.0, 6.3)	2.0(2.0, 4.3)	3.523^b^	0.172
AIS [score, M(Q1, Q3)]	0.0(0.0, 1.3)	1.0(0.0, 4.0)	0.5(0.0, 3.3)	2.484^b^	0.289
ESS [score, M(Q1, Q3)]	0.0(0.0, 1.0)	1.0(0.0, 2.0)	0.0(0.0, 1.0)	3.683^b^	0.159
Neuropsychological scale scores-cognitive assessment					
MMSE [score, M(Q1, Q3)]	28.0(28.0, 29.0)	24.0(23.0, 26.0) ^†^	16.0(12.0, 18.0) ^‡§^	75.522^b^	<0.001
MoCA (score, x̄ ± s)	26.47 ± 2.39	19.57 ± 3.87 ^†^	10.30 ± 4.34 ^‡§^	150.241^a^	<0.001
CDT [score, M(Q1, Q3)]	4.0(4.0, 4.0)	4.0(3.0, 4.0)	1.0(0.0, 3.0) ^‡§^	42.769^b^	<0.001
MES-T [score, M(Q1, Q3)]	44.0(40.8, 47.3)	40.5(32.8, 45.8) ^†^	23.0(11.0, 32.3) ^‡§^	38.487^b^	<0.001
MES-M [score, M(Q1, Q3)]	27.0(25.0, 28.3)	19.5(16.0, 23.0) ^†^	11.5(6.8, 13.0) ^‡§^	69.875^b^	<0.001
MES-E [score, M(Q1, Q3)]	43.0(36.8, 47.0)	27.5(20.8, 32.0) ^†^	11.5(6.8, 20.3) ^‡§^	64.873^b^	<0.001
AFT [score, M(Q1, Q3)]	19.5(16.0, 22.0)	13.5(11.3, 14.0) ^†^	9.0(6.0, 10.3) ^‡§^	53.681^b^	<0.001
BNT (score, x̄ ± s)	22.0(19.0, 26.0)	20.5(16.0, 23.0) ^†^	15.0(11.8, 18.0) ^‡§^	30.791^b^	<0.001
DST [score, M(Q1, Q3)]	13.0(10.0, 14.0)	11.0(9.0, 12.0) ^†^	7.5(5.0, 9.0) ^‡§^	46.859^b^	<0.001
FDST [score, M(Q1, Q3)]	8.0(6.8, 8.0)	7.0(6.0, 7.8) ^†^	5.0(3.0, 6.0) ^‡§^	36.080^b^	<0.001
BDST [score, M(Q1, Q3)]	5.0(4.0, 7.0)	4.0(3.0, 4.8) ^†^	2.0(2.0, 3.0) ^‡§^	45.898^b^	<0.001
DSST [score, M(Q1, Q3)]	33.0(31.0, 35.8)	22.0(17.0, 27.8)	5.5(0.0, 11.3) ^‡§^	65.637^b^	<0.001
Muscle loss related clinical microscopic indicators					
ASMI [kg/m^2^, M(Q1, Q3)]	7.2(6.8, 7.8)	7.0(6.2, 7.5)	6.1(5.7, 7.7) ^‡^	7.839^b^	0.020
Grip strength (kg, x̄ ± s)	29.84 ± 7.35	27.18 ± 8.08	22.86 ± 7.41 ^‡§^	6.414^a^	0.003
Chair stand test [s, M(Q1, Q3)]	9.9(7.7, 12.0)	11.0(9.1, 12.3)	11.5(9.9, 14.0) ^‡^	7.871^b^	0.020
Gait speed (s, x̄ ± s)	1.20 ± 0.29	1.45 ± 0.84	1.12 ± 0.31	2.948^a^	0.058
Calf circumference (cm, x̄ ± s)	36.45 ± 2.79	36.07 ± 2.85	34.88 ± 3.14	2.335^a^	0.103
SPPB [score, M(Q1, Q3)]	12.0(11.8, 12.0)	12.0(10.0, 12.0)	12.0(8.0, 12.0)	4.790^b^	0.091
Muscle loss related morphological microstructural indicators					
Brachialis					
Bra.MT [cm, M(Q1, Q3)]	1.5(1.5, 1.8)	1.4(1.3, 1.5) ^†^	1.3(1.0, 1.5) ^‡§^	22.225^b^	<0.001
Bra.CSA[cm^2^, M(Q1, Q3)]	7.8(7.2, 9.1)	6.2(5.1, 7.1) ^†^	4.8(3.8, 6.3) ^‡§^	31.784^b^	<0.001
Bra.YM (kPA, x̄ ± s)	30.33 ± 6.24	30.57 ± 6.64	29.87 ± 5.84	0.095^a^	0.909
Bra.MT/CSA [M(Q1, Q3)]	0.2(0.2, 0.2)	0.2(0.2, 0.3) ^†^	0.3(0.2, 0.3) ^‡§^	19.474^b^	<0.001
Biceps brachii					
Bic.MT [cm, M(Q1, Q3)]	2.1(1.9, 2.3)	1.8(1.6, 1.9) ^†^	1.7(1.4, 1.9) ^‡^	27.631^b^	<0.001
Bic.CSA [cm^2^, M(Q1, Q3)]	10.0(8.0, 12.1)	6.6(6.0, 8.1) ^†^	5.7(4.8, 8.1) ^‡^	29.311^b^	<0.001
Bic.YM [kPA, M(Q1, Q3)]	27.3(23.2, 31.4)	32.7(25.3, 44.9)	40.5(25.7, 59.6) ^‡^	8.022^b^	0.018
Bic.MT/CSA [M(Q1, Q3)]	0.2(0.2, 0.2)	0.3(0.2, 0.3) ^†^	0.3(0.2, 0.3) ^‡^	19.028^b^	<0.001
Gastrocnemius					
Gas.MT [cm, M(Q1, Q3)]	1.7(1.6, 1.9)	1.5(1.4, 1.7) ^†^	1.4(1.3, 1.5) ^‡^	20.723^b^	<0.001
Gas.CSA [cm^2^, M(Q1, Q3)]	9.5(8.1, 11.4)	7.4(6.4, 8.5) ^†^	6.6(5.4, 7.6) ^‡^	23.649^b^	<0.001
Gas.FL (cm, x̄ ± s)	4.64 ± 0.64	4.44 ± 0.72	4.49 ± 0.58	0.753^a^	0.474
Gas.PA [°, M(Q1, Q3)]	24.9(22.3, 26.6)	21.0(18.5, 25.5) ^†^	20.1(17.6, 21.9) ^‡§^	18.809^b^	<0.001
Gas.YM [kPA, M(Q1, Q3)]	12.2(11.3, 16.2)	13.5(10.4, 16.0)	11.9(10.0, 17.1)	0.149^b^	0.928
Gas.FL-C [cm, M(Q1, Q3)]	3.0(2.6, 3.1)	2.6(2.3, 2.9) ^†^	2.8(2.5, 3.3)	6.208^b^	0.045
Gas.PA-C (°, x̄ ± s)	37.60 ± 8.70	35.97 ± 8.02	30.43 ± 7.88 ^‡§^	6.242^a^	0.003
Gas.YM-C [kPA, M(Q1, Q3)]	84.7(66.3, 111.6)	90.5(55.8, 111.7)	51.4(41.3, 79.4) ^‡^	8.870^b^	0.012
Gas.FL-T (cm, x̄ ± s)	5.11 ± 0.81	4.77 ± 0.50	4.87 ± 0.63	2.011^a^	0.140
Gas.PA-T (°, x̄ ± s)	22.18 ± 2.84	19.91 ± 3.94^†^	19.64 ± 3.81 ^‡^	4.599^a^	0.013
Gas.YM-T [kPA, M(Q1, Q3)]	115.2(78.8, 139.5)	91.3(57.3, 136.5)	64.7(42.9, 101.7) ^‡^	10.985^b^	0.004
Gas.MT/CSA [M(Q1, Q3)]	0.2(0.2, 0.2)	0.2(0.2, 0.2) ^†^	0.2(0.2, 0.2) ^‡^	18.218^b^	<0.001
Gas.LF/MT [M(Q1, Q3)]	2.6(2.5, 2.8)	2.9(2.3, 3.4)	3.1(2.8, 3.4) ^‡^	11.102^b^	0.004
ΔPA (°, x̄ ± s)	12.95 ± 7.10	13.58 ± 7.32	10.33 ± 6.33	1.831^a^	0.167
ΔFL [cm, M(Q1, Q3)]	1.7(1.3, 2.0)	1.9(1.4, 2.2)	1.7(1.3, 2.0)	1.887^b^	0.389
ΔYM (kPA, x̄ ± s)	70.1(51.0, 97.1)	74.7(40.0, 98.8)	37.3(25.6, 68.3) ^‡^	8.707^b^	0.013
Serum VD level					
25OHD_3_ [ng/mL, M(Q1, Q3)]	27.4(22.2, 30.4)	21.6(16.4, 26.3) ^†^	15.7(11.9, 19.9) ^‡§^	30.236^b^	<0.001
25OHD [ng/mL, M(Q1, Q3)]	29.5(23.1, 31.6)	21.6(17.3, 26.3) ^†^	15.7(11.9, 19.9) ^‡§^	35.089^b^	<0.001
VDD[n (%)]	2(6.7)	10(35.7)	23(76.7)		

*^a^*Normally distributed variable is expressed as mean ± standard deviation and analyzed by ANOVA of three groups (*F* value).

^b^ Non-normally distributed variable is expressed as median (interquartile range) and analyzed by Kruskal–Wallis H-test of three groups (*H* value).

^c^ Categorical variables are expressed as frequency (percent; χ^2^ value).

† mild AD significantly different from NC(*P* <0.05);

‡ moderate AD significantly different from NC(*P* <0.05);

^§^ mild AD significantly different from moderate AD(*P* <0.05). AD, Alzheimer’s disease; NC, normal controls; CHD, coronary heart disease; BMI, body mass index; MNA, mini nutritional assessment; DDS, dietary diversity score. HAMD, Hamilton depression scale; HAMA, Hamilton anxiety scale; PSQI, Pittsburgh sleep quality index; AIS, Athens insomnia scale; ESS, Epworth sleepiness scale. MMSE, Mini-Mental State Examination; MoCA, Montreal cognitive assessment; CDT, clock drawing test; MES-T, memory and executive screening scale-total score, MES-M, memory and executive screening scale-memory factor score; MES-E, memory and executive screening scale-executive factor score; AFT, annimal fluent test; BNT, Boston naming test; DST, digital span test; FDST, forward digital span test; BDST, backward digital span test; DSST, digit symbol substitution test; ASMI, appendicular skeletal muscle mass index; SPPB, short physical performance battery; Bra, Brachialis; Bic, Biceps brachii; Gas. Gastrocnemius; MT, muscle thickness; CSA, muscle cross-sectional area; FL, fascicle length; PA, pennate angle; YM, Young’s modulus; FL-C, FL in contracted state; PA-C, PA in contracted state; YM-C, YM in contracted state; FL-T, FL in tensed state; PA-T, PA in tensed state; YM-T, YM in tensed state; ΔFL, the absolute value of the difference in FL between two muscle states; ΔPA, the absolute value of the difference in pennate angle between two muscle states; ΔYM, the absolute value of the difference in Young’s modulus between two muscle states; VD, vitamin D; 25OHD_3_, 25-hydroxyvitamin D_3_; 25OHD, 25-hydroxyvitamin D; VDD, vitamin D deficiency.

### 3.2 Correlation of VD level with cognitive function and muscle loss in patients with AD

Adjusted for age, sex, and BMI as confounders, serum 25OHD_3_ and 25OHD concentrations were positively correlated with MMSE, CDT, MoCA, MES-M, MES-T, AFT, DST, and DSST scores in the AD group (all *P* < 0.05).

**FIGURE 1:**
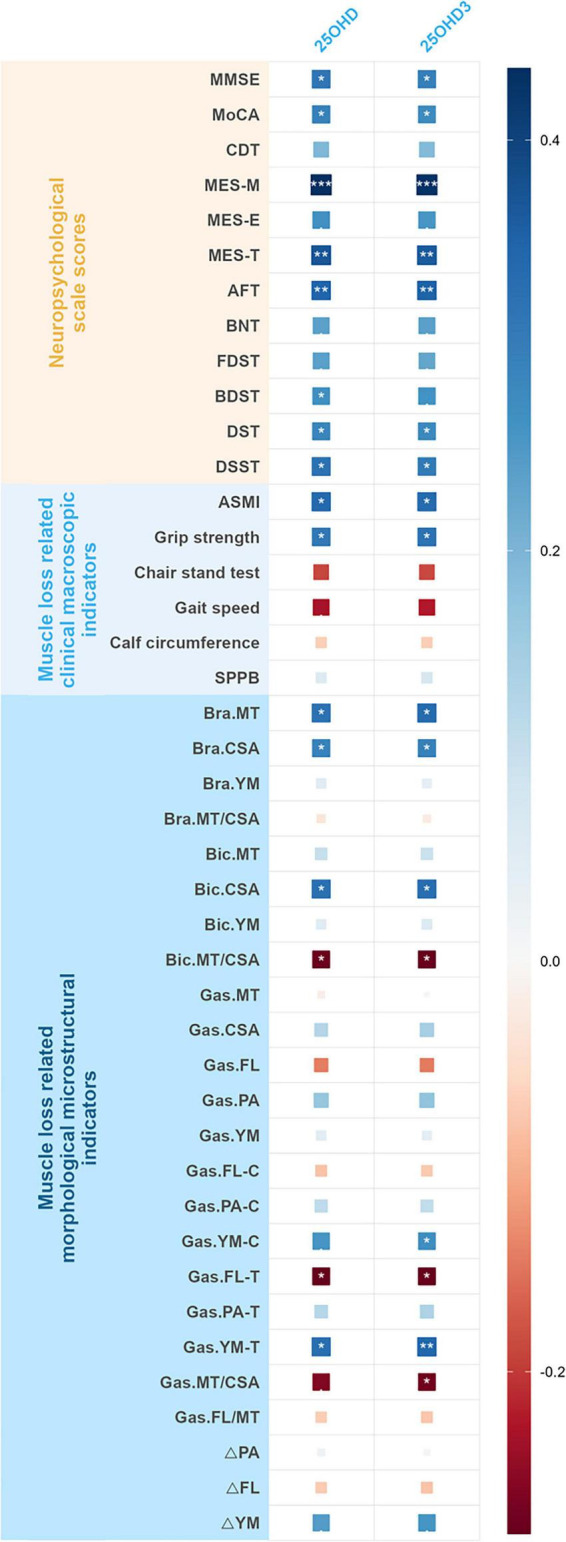
Heatmap illustrating the correlation of serum VD levels with cognitive function and muscle loss related indicators in patients with AD. *P* < 0.1, *P < 0.05, **P < 0.01, ***P < 0.001. AD, Alzheimer’s disease; VD, vitamin D; 25OHD3, 25-hydroxyvitamin D3; 25OHD, 25-hydroxyvitamin D; MMSE, Mini-Mental State Examination; MoCA, Montreal Cognitive Assessment; CDT, Clock Drawing Test; MES-T, Memory and Executive Screening Scale-Total score, MES-M, Memory and Executive Screening Scale-Memory factor score; MES-E, Memory and Executive Screening Scale-Executive factor score; AFT, Animal Fluent Test; BNT, Boston Naming Test; DST, Digital Span Test; FDST, Forward Digital Span Test; BDST, Backward Digital Span Test; DSST, Digit Symbol Substitution Test; ASMI, appendicular skeletal muscle mass index; SPPB, short physical performance battery; Bra. Brachialis; Bic. Biceps brachii; Gas. Gastrocnemius; MT, muscle thickness; CSA, muscle cross-sectional area; FL, fascicle length; PA, pennate angle; YM, Young’s modulus; FL-C, FL in contracted state; PA-C, PA in contracted state; YM-C, YM in contracted state; FL-T, FL in tensed state; PA-T, PA in tensed state; YM-T, YM in tensed state; ΔFL, the absolute value of the difference in FL between two muscle states; ΔPA, the absolute value of the difference in pennate angle between two muscle states; ΔYM, the absolute value of the difference in Young’s modulus between two muscle states.

Adjusted for age, sex, and BMI as confounders, in terms of clinical macroscopic indicators related to muscle loss, 25OHD_3_ and 25OHD concentrations correlated with ASMI and grip strength in patients with AD (all *P* < 0.05); in terms of morphological microstructural indicators related to muscle loss, serum 25OHD_3_ and 25OHD concentration correlated with bra.MT, bra.CSA, bra.MT/CSA, bic,CSA, bic.MT/CSA, gas.FL-T, gas.YM-T in AD groups (all *P* < 0.05). In addition, 25OHD_3_ levels were correlated with gas.YM-C, and gas.MT/CSA (all *P* < 0.05) (Figure 1 and Table 2).

**TABLE 2:** Correlation of serum VD levels with cognitive function and muscle loss related indicators in patients with AD.

Variable	25OHD		25OHD_3_	
	*r-*value	*P-*value	*r-*value	*P-*value
Neuropsychological scale scores				
MMSE	0.313	0.020	0.296	0.028
MoCA	0.293	0.030	0.273	0.044
CDT	0.196	0.151	0.191	0.162
MES-M	0.434	0.001	0.429	0.001
MES-E	0.264	0.052	0.255	0.060
MES-T	0.371	0.005	0.363	0.006
AFT	0.347	0.009	0.350	0.009
BNT	0.236	0.083	0.238	0.081
FDST	0.236	0.083	0.227	0.096
BDST	0.266	0.050	0.260	0.055
DST	0.285	0.035	0.277	0.040
DSST	0.322	0.016	0.305	0.024
Muscle loss related clinical microscopic indicators				
ASMI	0.339	0.011	0.335	0.012
Grip strength	0.309	0.022	0.319	0.018
Chair stand test	−0.190	0.166	−0.188	0.169
Gait speed	−0.230	0.091	−0.223	0.102
Calf circumference	−0.067	0.627	−0.068	0.620
SPPB	0.060	0.666	0.070	0.611
Muscle loss related morphological microstructural indicators				
Brachialis				
Bra.MT	0.322	0.016	0.336	0.012
Bra.CSA	0.290	0.032	0.293	0.030
Bra.YM	0.051	0.709	0.040	0.774
Bra.MT/CSA	−0.032	0.818	−0.022	0.876
Biceps brachii				
Bic.MT	0.099	0.473	0.095	0.489
Bic.CSA	0.326	0.015	0.329	0.014
Bic.YM	0.051	0.713	0.059	0.671
Bic.MT/CSA	−0.275	0.042	−0.277	0.040
Gastrocnemius				
Gas.MT	−0.017	0.904	−0.006	0.966
Gas.CSA	0.128	0.352	0.145	0.292
Gas.FL	−0.143	0.297	−0.145	0.293
Gas.PA	0.169	0.217	0.174	0.204
Gas.YM	0.048	0.730	0.041	0.767
Gas.FL-C	−0.080	0.564	−0.072	0.599
Gas.PA-C	0.113	0.412	0.108	0.430
Gas.YM-C	0.257	0.059	0.268	0.048
Gas.FL-T	−0.278	0.040	−0.277	0.041
Gas.PA-T	0.125	0.364	0.135	0.327
Gas.YM-T	0.330	0.014	0.343	0.010
Gas.MT/CSA	−0.258	0.057	−0.270	0.046
Gas.FL/MT	−0.069	0.618	−0.077	0.574
△PA	0.019	0.890	0.011	0.938
△FL	−0.071	0.606	−0.079	0.566
△YM	0.245	0.072	0.257	0.058

AD, Alzheimer’s disease; VD, vitamin D; 25OHD_3_, 25-hydroxyvitamin D_3_; 25OHD, 25-hydroxyvitamin D; MMSE, Mini-Mental State Examination; MoCA, Montreal cognitive assessment; CDT, clock drawing test; MES-T, memory and executive screening scale-total score, MES-M, memory and executive screening scale-memory factor score; MES-E, memory and executive screening scale-executive factor score; AFT, annimal fluent test; BNT, Boston naming test; DST, digital span test; FDST, forward digital span test; BDST, backward digital span test; DSST, digit symbol substitution test; ASMI, appendicular skeletal muscle mass index; SPPB, short physical performance battery; Bra, Brachialis; Bic, Biceps brachii; Gas. Gastrocnemius; MT, muscle thickness; CSA, muscle cross-sectional area; FL, fascicle length; PA, pennate angle; YM, Young’s modulus; FL-C, FL in contracted state; PA-C, PA in contracted state; YM-C, YM in contracted state; FL-T, FL in tensed state; PA-T, PA in tensed state; YM-T, YM in tensed state; ΔFL, the absolute value of the difference in FL between two muscle states; ΔPA, the absolute value of the difference in pennate angle between two muscle states; ΔYM, the absolute value of the difference in Young’s modulus between two muscle states.

### 3.3 Correlation between cognitive function and muscle loss related indicators in patients with AD

**FIGURE 2:**
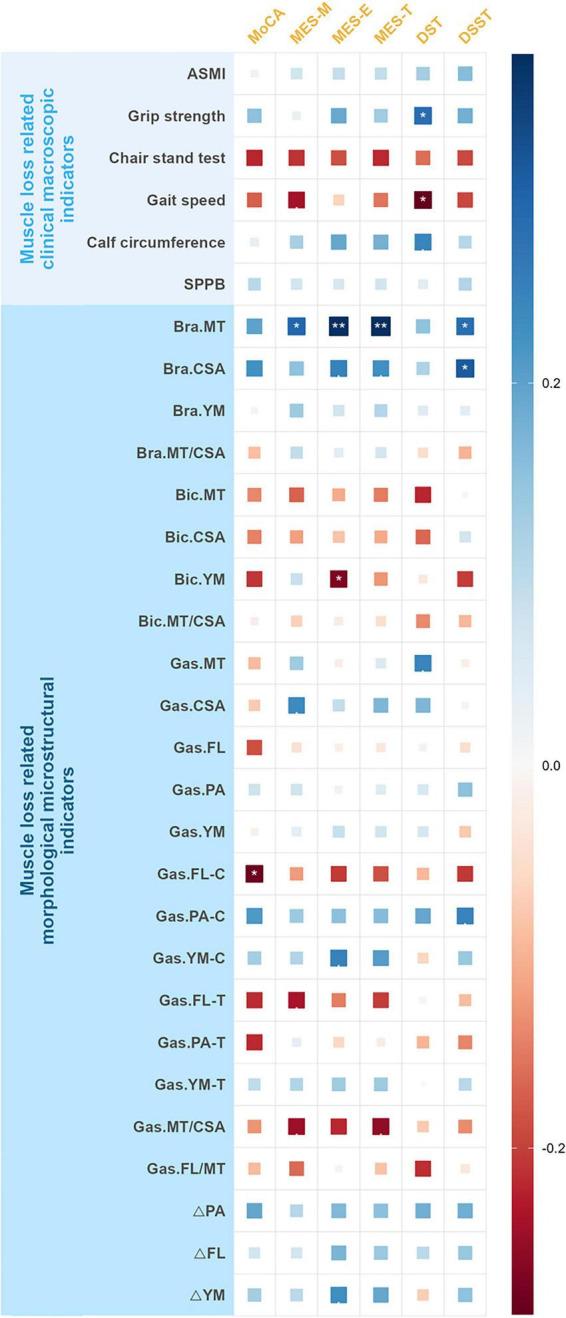
Heatmap showing the association between cognitive function and muscle loss related indicators in patients with AD. *P* < 0.1, *P < 0.05, **P < 0.01, ***P < 0.001. AD, Alzheimer’s disease; MMSE, Mini-Mental State Examination; MoCA, Montreal Cognitive Assessment; CDT, Clock Drawing Test; MES-T, Memory and Executive Screening Scale-Total score, MES-M, Memory and Executive Screening Scale-Memory factor score; MES-E, Memory and Executive Screening Scale-Executive factor score; AFT, Animal Fluent Test; BNT, Boston Naming Test; DST, Digital Span Test; FDST, Forward Digital Span Test; BDST, Backward Digital Span Test; DSST, Digit Symbol Substitution Test; ASMI, appendicular skeletal muscle mass index; SPPB, short physical performance battery; Bra. Brachialis; Bic. Biceps brachii; Gas. Gastrocnemius; MT, muscle thickness; CSA, muscle cross-sectional area; FL, fascicle length; PA, pennate angle; YM, Young’s modulus; FL-C, FL in contracted state; PA-C, PA in contracted state; YM-C, YM in contracted state; FL-T, FL in tensed state; PA-T, PA in tensed state; YM-T, YM in tensed state; ΔFL, the absolute value of the difference in FL between two muscle states; ΔPA, the absolute value of the difference in pennate angle between two muscle states; ΔYM, the absolute value of the difference in Young’s modulus between two muscle states.

Adjusted for age, sex, and BMI as confounders, in terms of clinical macroscopic indicators related to muscle loss, DST scores correlated with grip strength and gait speed in patients with AD (all *P* < 0.05); in terms of morphological microstructural indicators, MoCA correlated with gas.FL-C, in addition, MES-M, MES-E, MES-T, and DSST correlated with bra.MT, as well as DSST correlated with bra.CSA, and MES-E correlated with bic.YM (all *P* < 0.05) (Figure 2 and Table 3).

**TABLE 3:** Correlation between cognitive function and muscle loss related indicators in patients with AD.

Variable	MoCA		MES-M		MES-E		MES-T		DST		DSST	
	*r*-value	*P*-value	*r*-value	*P*-value	*r*-value	*P*-value	*r*-value	*P*-value	*r*-value	*P*-value	r 值	*P* 值
Muscle loss related clinical microscopic indicators												
ASMI	0.015	0.911	0.075	0.588	0.087	0.529	0.090	0.515	0.127	0.354	0.160	0.243
Grip strength	0.153	0.265	0.026	0.851	0.190	0.165	0.131	0.342	0.282	0.037	0.180	0.189
Chair stand test	−0.221	0.105	−0.212	0.120	−0.187	0.171	−0.218	0.110	−0.160	0.244	−0.192	0.161
Gait speed	−0.172	0.210	−0.240	0.077	−0.064	0.643	−0.154	0.261	−0.286	0.034	−0.193	0.158
Calf circumference	0.030	0.827	0.122	0.373	0.191	0.162	0.177	0.196	0.245	0.071	0.106	0.440
SPPB	0.102	0.461	0.069	0.617	0.061	0.656	0.071	0.606	0.040	0.772	0.110	0.422
Muscle loss related morphological microstructural indicators												
Brachialis												
Bra.MT	0.199	0.145	0.294	0.029	0.370	0.005	0.371	0.005	0.150	0.273	0.282	0.037
Bra.CSA	0.224	0.101	0.152	0.269	0.249	0.066	0.227	0.095	0.116	0.399	0.311	0.021
Bra.YM	0.009	0.950	0.134	0.328	0.072	0.599	0.109	0.428	0.045	0.747	0.040	0.774
Bra.MT/CSA	−0.087	0.526	0.093	0.497	0.037	0.791	0.067	0.626	−0.049	0.724	−0.099	0.470
Biceps brachii												
Bic.MT	−0.138	0.316	−0.169	0.216	−0.107	0.436	−0.147	0.283	−0.221	0.105	−0.003	0.985
Bic.CSA	−0.144	0.296	−0.119	0.388	−0.082	0.551	−0.108	0.434	−0.165	0.229	0.071	0.607
Bic.YM	−0.208	0.128	0.083	0.545	−0.267	0.048	−0.127	0.356	−0.029	0.832	−0.203	0.138
Bic.MT/CSA	−0.016	0.910	−0.067	0.628	−0.025	0.857	−0.047	0.732	−0.136	0.323	−0.094	0.497
Gastrocnemius												
Gas.MT	−0.090	0.516	0.132	0.338	−0.017	0.901	0.052	0.706	0.246	0.070	−0.018	0.896
Gas.CSA	−0.073	0.595	0.235	0.084	0.090	0.515	0.168	0.221	0.169	0.218	0.008	0.953
Gas.FL	−0.187	0.172	−0.042	0.759	−0.017	0.900	−0.031	0.822	0.014	0.922	−0.047	0.732
Gas.PA	0.077	0.575	0.075	0.587	0.016	0.909	0.045	0.742	0.059	0.668	0.153	0.263
Gas.YM	−0.013	0.924	0.038	0.785	0.085	0.537	0.071	0.607	0.060	0.661	−0.076	0.582
Gas.FL-C	−0.282	0.037	−0.122	0.375	−0.205	0.132	−0.186	0.174	−0.093	0.502	−0.206	0.131
Gas.PA-C	0.214	0.117	0.136	0.324	0.154	0.262	0.160	0.242	0.192	0.161	0.247	0.069
Gas.YM-C	0.127	0.357	0.110	0.423	0.249	0.067	0.208	0.128	−0.061	0.660	0.140	0.308
Gas.FL-T	−0.217	0.112	−0.236	0.083	−0.147	0.286	−0.203	0.136	0.007	0.957	−0.088	0.524
Gas.PA-T	−0.218	0.109	0.031	0.823	−0.059	0.667	−0.022	0.872	−0.096	0.486	−0.140	0.308
Gas.YM-T	0.094	0.496	0.110	0.425	0.133	0.332	0.135	0.325	0.000	0.998	0.103	0.455
Gas.MT/CSA	−0.128	0.350	−0.245	0.072	−0.219	0.108	−0.253	0.062	−0.074	0.590	−0.135	0.327
Gas.FL/MT	−0.090	0.512	−0.163	0.235	−0.009	0.951	−0.083	0.548	−0.215	0.114	−0.030	0.829
△PA	0.194	0.156	0.106	0.440	0.166	0.227	0.154	0.262	0.181	0.187	0.182	0.183
△FL	0.071	0.607	0.069	0.616	0.171	0.212	0.139	0.310	0.099	0.474	0.142	0.303
△YM	0.127	0.355	0.102	0.458	0.231	0.090	0.192	0.160	−0.071	0.609	0.151	0.270

AD, Alzheimer’s disease; MoCA, Montreal cognitive assessment; MES-T, memory and executive screening scale-total score, MES-M, memory and executive screening scale-memory factor score; MES-E, memory and executive screening scale-executive factor score; DST, digital span test; DSST, digit symbol substitution test; ASMI, appendicular skeletal muscle mass index; SPPB, short physical performance battery; Bra. Brachialis; Bic. Biceps brachii; Gas. Gastrocnemius; MT, muscle thickness; CSA, muscle cross-sectional area; FL, fascicle length; PA, pennate angle; YM, Young’s modulus; FL-C, FL in contracted state; PA-C, PA in contracted state; YM-C, YM in contracted state; FL-T, FL in tensed state; PA-T, PA in tensed state; YM-T, YM in tensed state; ΔFL, the absolute value of the difference in FL between two muscle states; ΔPA, the absolute value of the difference in pennate angle between two muscle states; ΔYM, the absolute value of the difference in Young’s modulus between two muscle states.

### 3.4 Mediating effects of VD level between muscle loss and cognitive function in patients with AD

**FIGURE 3:**
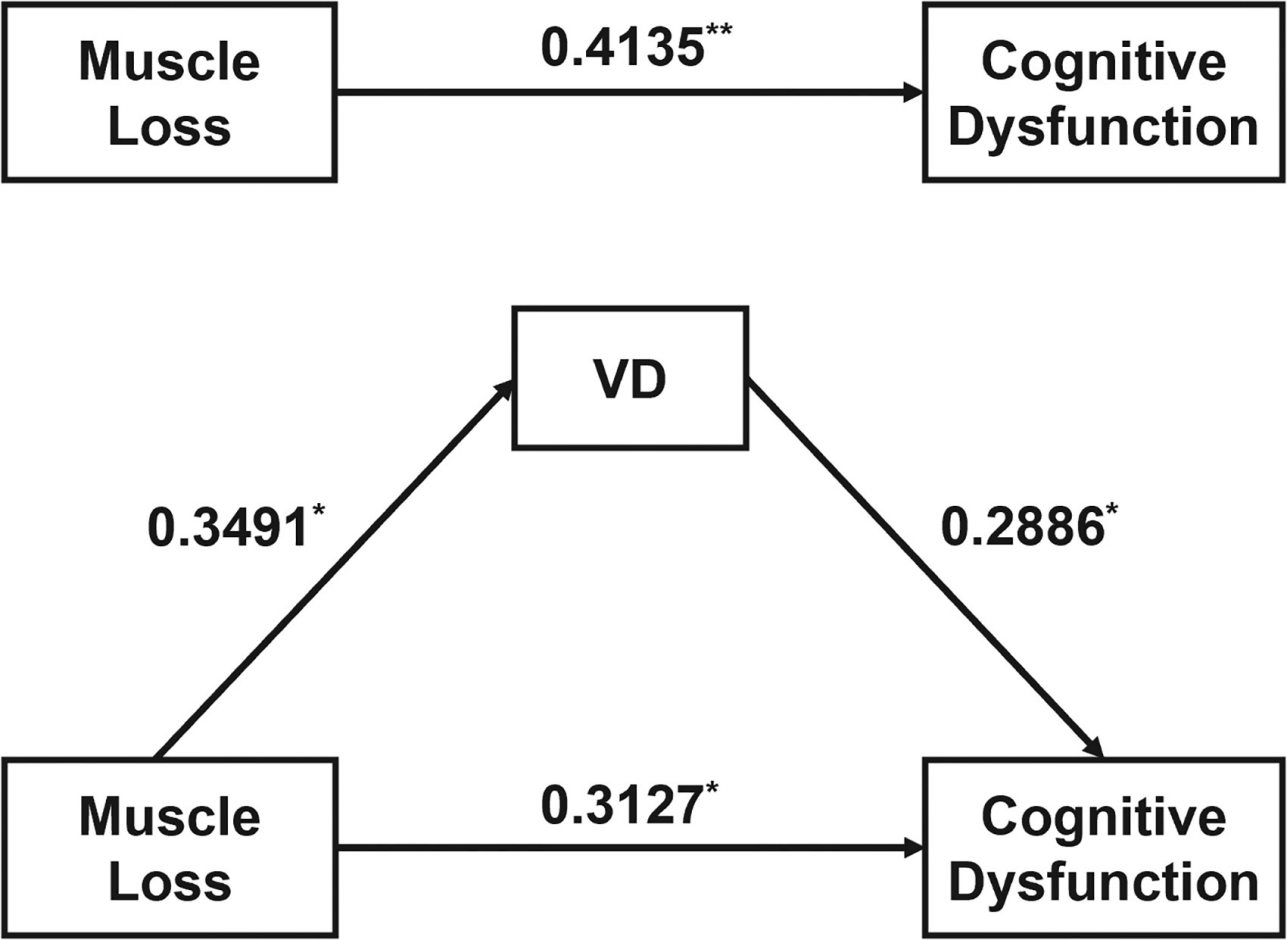
Mediation analysis of serum VD level on the relationship between muscle loss and cognitive function in patients with AD. *P < 0.05, **P < 0.01.

Age, sex, and BMI were included as covariates, bra.MT was served as the independent variable (X), MES-T as the dependent variable (Y), and serum 25OHD concentration as the mediator (M), and continuous variables were z-score standardized. Three regression models were constructed for X →regre →regression → Y, respectively, and the variance inflation factor (VIF) of the variables in each model were calculated separately, and all VIF < 2, suggesting no multicollinearity issues. The results indicated that, after controlling covariates, the total effect regression showed that bra.MT had a statistically significant impact on MES-T (β = 0.4135, 95% CI 0.1280–0.6989, *P* < 0.01), and this effect remained significant when accounting for 25OHD (β = 0.3127, 95% CI 0.0208–0.6054, *P* < 0.05). Moreover, bra.MT significantly influenced 25OHD (β = 0.3491, 95% CI 0.0665–0.6318, *P* < 0.05) (Figure 3 and Table 4).

**TABLE 4:** Regression analysis between variables.

Variable	MES-T	25OHD	MES-T
Bra.MT	0.4135** (0.1280, 0.6989)	0.3491* (0.0665, 0.6318)	0.3127* (0.0208, 0.6045)
Age	−0.1210 (−0.3741, 0.1322)	−0.1475 (−0.3982, 0.1032)	−0.0784 (−0.3266, 0.1699)
Male	−0.0769 (−0.6369, 0.4830)	0.3343 (−0.2202, 0.8888)	−0.1734 (−0.7229, 0.3760)
BMI	0.1053 (−0.1489, 0.3595)	−0.2370 (−0.4887, 0.0147)	0.1737 (−0.0804, 0.4279)
25OHD	–	–	0.2886* (0.0193, 0.5579)
*F*	3.9746	4.3161**	4.3220
*R*	0.4804	0.4957	0.5418
*R* ^2^	0.2308	0.2457	0.2936

95% confidence intervals in brackets. *P < 0.05, **P < 0.01. AD, Alzheimer’s disease; VD, vitamin D; 25OHD, 25-hydroxyvitamin D; MES-T, memory and executive screening scale-total score; Bra. Brachialis; MT, muscle thickness.

Mediation analysis using Bootstrap sampling test showed that muscle loss (bra.MT) had a direct effect on cognitive function (MES-T scores) of 0.3127 (95% CI 0.0208–0.6045, *P* < 0.05), and a total effect of 0.4135 (95% CI 0.1280–0.6989, *P* < 0.05). The indirect effect of serum VD levels (25OHD) on cognitive function (MES-T score) was 0.1008 (95% CI 0.0132–0.2446), with the confidence interval for the indirect effect excluding 0, indicating significant mediation. All paths were significant, and the mediation model results were robust (Table 5).

**TABLE 5:** Mediation analysis of serum VD level on the relationship between muscle loss and cognitive function in patients with AD.

Paths	β	LLCI	ULCI
Total effect	0.4135**	0.1280	0.6989
Direct effect	0.3127*	0.0208	0.6045
Indirect effect	0.1008	0.0132	0.2446

β, Observed Coefficient; LLCI, lower level for confidence interval; ULCI, upper level for confidence interval. *P < 0.05, **P < 0.01.

## 4 Discussion

This study aimed to establish a multidimensional assessment framework for VD level, muscle loss, and cognitive function in patients with AD, analyze their interrelations, and investigate the potential role of VD in muscle loss and cognitive function. The results were (1) Significant differences in cognitive domains, muscle loss related clinical macroscopic and morphological microstructural indicators, and serum VD levels among mild and moderate AD and NC group. (2) Correlations among serum VD levels, muscle loss related indicators (notably muscle mass and upper limb strength), and broad cognitive domains. (3) VD level partially mediates the link between muscle loss, particularly muscle mass reduction, and cognitive dysfunction. This suggests that the multidimensional assessment of muscle loss should be emphasized and that effective interventions should be promptly implemented for VDD in the early diagnosis and treatment of patients with AD. This study suggests implementing regular physical exercise and scientific dietary nutrition to prevent muscle loss and VDD, which may potentially be associated with a reduced risk of AD and slower cognitive decline, offering great social value.

### 4.1 Co-occurrence of muscle loss and VDD in AD progression

Many epidemiological data showed that in addition to the characteristic cognitive dysfunction, patients with AD are often associated with age-related changes such as muscle loss and VDD. For instance, a meta-analysis of 27 studies found that the prevalence of muscle loss in patients with AD was about 33.9%, which was significantly higher than that in the healthy older adults (Su et al., 2025). Ogawa et al. assessed 285 patients with AD and healthy controls, revealing higher muscle loss prevalence in early-stage AD, which escalated significantly with disease progression (Ogawa et al., 2018). A meta-analysis of 284 studies confirmed that VD levels were generally lower in patients with AD compared to controls (Annweiler et al., 2013). This study demonstrated statistically significant differences in cognitive domains, muscle loss related indicators, and serum VD levels among mild and moderate AD groups and the NC group (*P* < 0.05). As the degree of cognitive dysfunction progressed in patients with AD, serum 25OHD concentrations decreased and muscle loss worsen, which is consistent with the previous studies mentioned. The mechanism may be due to the overlapping effects of aging-related pathways shared by cognitive dysfunction, muscle atrophy, and VDD in patients with AD. Inflammaging, a core mechanism of aging, may contributes to these overlapping effects through multiple pathways (Zordoky and El-Kadi, 2009; Oudbier et al., 2022; Ma et al., 2025). Specifically, inflammaging accelerates muscle protein breakdown via the ubiquitin-proteasome system (UPS), leading to muscle fiber atrophy and muscle loss (Oudbier et al., 2022), while simultaneously promoting brain Aβ deposition and Tau protein aggregation (Ma et al., 2025). The NF-κB pathway, a critical mediator of inflammaging, affect the synthesis of active VD through the regulation of CYP27B1 (Zordoky and El-Kadi, 2009). In turn, the pathological progression of AD accelerates aging and intensifies age-related pathological changes, thereby exacerbating the overlapping effects. For instance, studies have indicated that neurons expressing the senescence marker p16^INK4A^ are more common in AD brains than in normally aging brains. The characteristic AD pathologies, Aβ deposition and Tau protein aggregation, can activate the mTOR pathway to aggravate autophagy deficits, while deposited Aβ triggers NF-κB, IRFs, and activates the NLRP3 inflammasome to worsen inflammaging (Ma et al., 2025). All above findings suggest a clinical linkage among cognitive dysfunction, muscle loss, and VDD in patients with AD. As AD progresses, both muscle loss and VDD exacerbate to varying degrees, which deserves more attention.

### 4.2 Multimodal ultrasound assessing morphological changes beyond clinical muscle loss in AD

In recent years, muscle loss in patients with AD has become a research hotspot, and most of the research focuses on the use of comprehensive clinical assessment tools to evaluate muscle loss related clinical macroscopic indicators, such as grip strength, ASMI, etc. In particular, grip strength effectively assessed muscle strength of upper limb, ASMI represented muscle mass, and gait speed assessed physical performance. These macroscopic indicators focus on the early clinical screening and risk prediction of patients with AD, while neglecting the microscopic changes in the architecture and function of specific muscles. Multimodal muscle ultrasound, as an economic, convenient, safe, non-invasive, and high-resolution emerging technology, can accurately evaluate muscle morphology in real time, dynamically assess muscle structure, and indirectly reflect the changes in muscle function, so as to effectively identify the potential muscle loss population at an early stage.

In this study, we integrate muscle loss related clinical macroscopic indicators and morphological microstructural indicators to visualize targeted skeletal muscle loss from macroscopic phenomenon to morphological microstructural changes. The results showed that, in terms of clinical macroscopic indicators, the moderate AD group had lower ASMI, grip strength and longer Chair stand test time than the NC group (all *P* < 0.05), which is consistent with previous meta-analysis that summarized several studies showing that clinical macroscopic indicators related to muscle loss, such as ASMI, grip strength, and gait speed, are poorer in patients with AD (Su et al., 2025). In terms of morphological microstructural indicators, there were significant differences in MT and CSA of the limbs and PA differing among the three groups. Specifically, MT, CSA, PA, FL and their changes with passive movement and force load are important objective indicators of muscle structure and function.MT and CSA can quantify muscle size, predict muscle mass and are closely related to muscle strength. CSA represents the maximum number of myosin bridges that can be activated in parallel during contraction and is a determinant of maximal muscle strength (Jones et al., 2008), PA reflects the arrangement of muscle fibers that also affects maximal muscle strength, and PA in different states of the muscle is closely related to the efficiency of force output (Van Hooren et al., 2024). Although there are not many similar studies investigating muscle loss in patients with AD from a morphological point of view, it has also been reported that patients with dementia have a lower MT of the gastrocnemius muscle compared to NC (Ülger et al., 2022), which supports this study. All above results suggest that muscle loss increases in patients with AD with the aggravation of the disease, and that a combined and precise assessment from the clinical macroscopic to the morphological microscopic can more effectively reflect the degree of muscle loss in patients with AD.

### 4.3 Associations between VD level, muscle loss, and cognitive dysfunction in AD

To further analyze the association among muscle loss, VD level and cognitive function, and to explore the interaction network and multidimensional regulatory system among the three in patients with AD, this study adjusted the age, sex and BMI for correlation analysis. This result showed that muscle mass and muscle strength of upper limbs, serum VD levels, and broad cognitive domains were significantly correlated in patients with AD. Previous cross-sectional studies have confirmed a correlation between muscle mass, muscle strength, and cognitive function (Su et al., 2025). From a clinical macroscopic perspective, our team’s previous studies indicated that muscle mass, muscle strength, and physical performance in patients with AD at different stages are associated with broad cognitive impairment (Liu et al., 2022). Furthermore, several studies have analyzed this correlation from the perspective of muscle morphology, for instance, low MT frequency of gastrocnemius muscle is independently associated with dementia including AD (Ülger et al., 2022). Recent reports suggest that temporalis muscle MT (Borda et al., 2024), bunion muscle MT (Varan et al., 2024), etc. are also closely associated with AD. In this regard, the “muscle-brain” axis hypothesis provides a key perspective for understanding the pathophysiological mechanisms underlying the association between muscle loss and cognitive function. These mechanisms involve myokines such as irisin, neurotrophic factors like BDNF, pro-inflammatory cytokines like IL-6, further encompassing neuromuscular junctions (NMJs), the UPS system, and the IGF-1 pathway, etc. (Pedersen, 2019; Oudbier et al., 2022; Arosio et al., 2023).

Multidimensionally focusing on muscle loss, we combined clinical macroscopic and morphological microscopic indicators, and our findings revealed that DST scores in the AD group correlated with ASMI and gait speed; MoCA in the AD group correlated with gas.FC-C, MES-M, MES-E, MES-T, and DSST correlated with bra.MT, and DSST also correlated with bra.CSA. MES-E correlated with bic.YM (all *P* < 0.05). These results collectively indicate that morphological microstructural indices exhibit significant and more extensive correlations with cognitive function and VD levels than clinical macroscopic indicators. This suggests that microstructural indicators provide superior sensitivity for evaluating muscle structure and localized metabolic changes. Despite the evaluation method of macroscopic indicators is simple, convenient and easy to operate, they are highly susceptible to various uncontrollable factors. In contrast, microstructural indicators can reflect muscle structure and metabolic changes earlier, potentially occurring before the decline of clinical macroscopic indicators. However, this is required to be confirmed by further longitudinal studies. Furthermore, this study found that microstructural indicators of the upper limb muscle strength exhibited a more significant correlation with cognitive function than the gastrocnemius muscle of the lower limb. A plausible explanation for this correlation is that fine motor skills of the upper limbs, such as writing, require sophisticated coordination between the cerebellum and the parietal cortex. This neurological link suggests that upper limb muscles, particularly the bra.MT, may be more representative of overall muscle mass decline. However, previous studies have found a correlation between lower limb muscle strength and cognitive function (Chen et al., 2022), which was inconsistent with our findings. Such inconsistencies could be attributed to several factors. First, the subjects of the present study were from the Chinese population, where a high cultural weighting of upper limb fine motor activity in daily life might influence the results. Second, the expression pattern of APOE ε4

allele is various across populations, which affects the sensitivity of the “muscle-brain” axis (Abondio et al., 2019; Abondio et al., 2023). Third, the muscle loss is affected by the different sex composition (Lu et al., 2023). Furthermore, the sensitivity and representativeness of the gastrocnemius muscle are limited, and the statistical power is affected due to the limitation of the sample size. Regarding the relationship between VD level, muscle loss and cognitive dysfunction in patients with AD, there have been numerous reports showing the correlation among the three and these findings are supported by evidence from mechanism studies. In particular, VD mediates a variety of biological effects through VDR, which is widely expressed not only in skeletal muscle cells, but also in most regions of the brain, especially in the hippocampus, the cortex, the hypothalamus and other regions closely related to cognitive function (Annweiler et al., 2015). The active form of VD also enhances the increased expression of trophic neuroproteins in the hippocampus and other areas of the brain to promote the synthesis of BNDF and regulate the genetic expression of a variety of neurotransmitters (Shea et al., 2023). It has been found that VD can be significantly associated with AD-related morphological changes in the brain by both modulating VDD and muscle loss. As early as 2014, a systematic review and analysis showed that VD depletion was associated with lower total brain volume (Annweiler et al., 2014); another study confirmed that 25OHD concentration was associated with total brain volume, white matter volume, gray matter volume change, white matter hyperintensities and other brain morphological indicators (Navale et al., 2022); and our team previous studies found that muscle loss related indicators in patients with AD at different clinical stages included muscle mass, muscle strength, physical performance are correlated with brain morphological indicators such as lateral temporal lobe atrophy, brain gray matter volume changes, brain white matter hyperintensities, brain white matter volume, etc., while these brain morphological indicators have been proved to be closely related to cognitive function (Weng et al., 2023; Liu R. et al., 2024; Liu S. W. et al., 2024).

### 4.4 VD as a potential factor connecting cognitive dysfunction and muscle loss

Current research supports a direct effect of muscle loss on cognitive function in patients with AD. A Mendelian Randomization (MR) study using clinical indicators related to muscle loss showed an independent causal effect of muscle loss on the risk of AD (Ye et al., 2023). Longitudinal studies have shown that healthy older adults with muscle loss experience faster cognitive decline in the last 6 years and are 60% more likely to develop AD (Moradi et al., 2024). Meta-analyses have found a 197% increase in the prevalence of AD due to muscle loss (Amini et al., 2024). A multi-cohort study over 28 years of longitudinal study showed that the average decline in exercise capacity in older adults, particularly hand strength, preceded cognitive decline (Oveisgharan et al., 2024). Animal studies have shown that skeletal muscle specific knockout of the myokine irisin gene in AD is associated with an increased prevalence of AD of 197%. Irisin gene in AD model mice exhibited reduced expression of PSD-95, a marker of synaptic plasticity in the hippocampus, accompanied by an amount of Aβ-42 deposition, whereas exogenous irisin intervention improved cognitive function in mice by activating the AMPK/BDNF pathway. Analysis of muscle-secreted myokines, including irisin and BDNF, can improve the expression of synaptic plasticity-related proteins, such as PSD-95 and Synapsin-1, via the “muscle-brain” axis, thus affecting cognitive function (Arosio et al., 2023). Resistance exercise has also been shown to increase prefrontal cortex and hippocampal activity by upregulating myokine expression through muscle contraction (Gonzalez-Gomez et al., 2026). In addition, there are also a large number of studies supporting the effect of VDD on cognitive dysfunction in patients with AD, with one MR finding that natural logarithmic conversion of 1-SD of 25OHD reduced the risk of AD by 25% (95% CI 1.03–1.51, *P* < 0.05), supporting a causal relationship between VDD and AD (Navale et al., 2022). Data from overseas longitudinal studies show a 40% reduction in AD incidence in healthy populations after moderate VD supplementation (Ghahremani et al., 2023). Data from a longitudinal study in China showed a significant reduction of 46.8% in AD risk after VD supplementation in healthy populations, and a 60.5% reduction in AD incidence after VD supplementation in populations with baseline cognitive dysfunction ^(^ Jiang et al., 2023). Mechanistic studies have observed changes in mRNA levels of genes related to Aβ processing in VDD-induced 5xF AD mice, leading to increased Aβ load and cognitive dysfunction in the brains of AD mice, and VD supplementation can improve pathological Aβ deposition in AD mice (Kang et al., 2022). To further clarify the possible role of VD level in the association between muscle loss and cognitive function in patients with AD, this study selected MES-T from the neuropsychological scales which assessed comprehensive cognitive domains, and bra.MT from muscle loss related morphological microstructure indicators, then combined with the 25OHD to perform the mediation effect analysis. The results demonstrated that the effects of muscle loss, especially muscle mass reduction, on cognitive function in patients with AD were statistically significant (β = 0.4135, *P* < 0.01), which remained statistically significant when the effect of VD levels was considered (β = 0.0.3127, *P* < 0.05). Even after considering the influence of VD levels, the impact remained significant (β = 0.3127, *P* < 0.05). Furthermore, VD deficiency partially mediated the effect of muscle loss on cognitive function (β = 0.1008, *P* < 0.05). Although we have not conducted specific experimental laboratory studies on this mediating mechanism, existing literature suggests that this mechanism might involve VD regulation of the Agrin/Lrp4/MuSK/Dok7 signaling pathway, which affects the aggregation of acetylcholine receptors (AChR) and the formation of neuromuscular junctions. This pathway might influence the “muscle-brain” axis, further linking muscle health and brain function (Arosio et al., 2023).

### 4.5 Limitations

This study still has some limitations: ➀The study selected the subjects in a single center and included a relatively small sample size, which limited the representativeness of the sample and might have a selection bias. The correlation of the indicators was not uniform. Future studies will expand the sample size, conduct multicenter joint studies, and include more diverse patient cohorts to increase the robustness and generalizability of the findings. In particular, more granular subgroup analyses are warranted. ➁Our findings remain preliminary and exploratory in nature. Due to the statistical approach employed, there is an inherent risk of multiple comparison bias, which may increase the potential for Type I errors. ➂There is a more complex interaction network among VD level, muscle loss, and cognitive function, and this study only built a simple mediation analysis using few indicators. In the future, we will further expand the sample size, conduct chain mediation analysis or other structural equation models to explore the mechanisms from various aspects. ➃The present study is a cross-sectional study, which is not able to monitor the dynamic effect of VD level on the association between muscle loss and cognitive function in patients with AD and clarify the causal relationship. In the future, we will further carry out longitudinal studies or GWAS-related MR causality analyses and basic laboratory research on specific mediating mechanisms. ➄We did not adjust for the season of sample collection. Vitamin D levels exhibit seasonal variation, and this unaccounted-for factor could introduce variability into the VD measurements and potentially bias its associations with other variables. ➅We did not adjust for bone mineral density. Although individuals with fracture or severe osteoarthrosis history were excluded, osteoporosis shares common risk factors with muscle loss and VDD and may confound the observed associations. Future studies should include bone mineral density as a confounder.

## 5 Conclusion

This study suggested that VD level, muscle loss and cognitive function in patients with AD are closely related, and that VD may partially mediate the association between muscle loss, especially muscle mass loss, and cognitive function. These results provide preliminary evidence for AD clinical management that could potentially benefit from comprehensive nutritional-exercise-cognitive assessment and joint intervention.

## Data Availability

The raw data supporting the conclusions of this article will be made available by the authors, without undue reservation.

## References

[B1] Abondio P. Bruno F. Luiselli D. (2023). Apolipoprotein E (APOE) haplotypes in healthy subjects from worldwide macroareas: a population genetics perspective for cardiovascular disease, neurodegeneration, and dementia. *Curr. Issues Mol. Biol*. 45 2817–2831. 10.3390/cimb45040184 37185708 PMC10137191

[B2] Abondio P. Sazzini M. Garagnani P. Boattini A. Monti D. Franceschi C. et al. (2019). The genetic variability of APOE in different human populations and its implications for longevity. *Genes* 10:222. 10.3390/genes10030222 30884759 PMC6471373

[B3] Amini N. Ibn Hach M. Lapauw L. Dupont J. Vercauteren L. Verschueren S. et al. (2024). Meta-analysis on the interrelationship between sarcopenia and mild cognitive impairment, Alzheimer’s disease and other forms of dementia. *J. Cachexia Sarcopenia Muscle* 15 1240–1253. 10.1002/jcsm.13485 38715252 PMC11294028

[B4] Annweiler C. Annweiler T. Montero-Odasso M. Bartha R. Beauchet O. (2014). Vitamin D and brain volumetric changes: systematic review and meta-analysis. *Maturitas* 78 30–39. 10.1016/j.maturitas.2014.02.013 24674855

[B5] Annweiler C. Dursun E. Féron F. Gezen-Ak D. Kalueff A. V. Littlejohns T. et al. (2015). ‘Vitamin D and cognition in older adults’: updated international recommendations. *J. Intern. Med*. 277 45–57. 10.1111/joim.12279 24995480

[B6] Annweiler C. Llewellyn D. J. Beauchet O. (2013). Low serum vitamin D concentrations in Alzheimer’s disease: a systematic review and meta-analysis. *J. Alzheimers Dis*. 33 659–674. 10.3233/JAD-2012-121432 23042216

[B7] Arosio B. Calvani R. Ferri E. Coelho-Junior H. J. Carandina A. Campanelli F. et al. (2023). Sarcopenia and cognitive decline in older adults: targeting the muscle-brain axis. *Nutrients* 15:1853. 10.3390/nu15081853 37111070 PMC10142447

[B8] Beeri M. S. Leugrans S. E. Delbono O. Bennett D. A. Buchman A. S. (2021). Sarcopenia is associated with incident Alzheimer’s dementia, mild cognitive impairment, and cognitive decline. *J. Am. Geriatr. Soc.* 69 1826–1835. 10.1111/jgs.17206 33954985 PMC8286176

[B9] Borda M. G. Patricio Baldera J. Patino-Hernandez D. Westman E. Pérez-Zepeda M. U. Tarazona-Santabalbina F. J. et al. (2024). Temporal muscle thickness predicts mortality and disability in older adults diagnosed with mild dementia. *J. Frailty Aging* 13 441–447. 10.14283/jfa.2024.39 39574265

[B10] Burtscher J. Millet G. P. Place N. Kayser B. Zanou N. (2021). The muscle-brain axis and neurodegenerative diseases: the key role of mitochondria in exercise-induced neuroprotection. *Int. J. Mol. Sci*. 22:6479. 10.3390/ijms22126479 34204228 PMC8235687

[B11] Caballero-García A. Córdova-Martínez A. Vicente-Salar N. Roche E. Pérez-Valdecantos D. (2021). Vitamin D, its role in recovery after muscular damage following exercises. *Nutrients* 13:2336. 10.3390/nu13072336 34371846 PMC8308579

[B12] Chen L.-K. Woo J. Assantachai P. Auyeung T.-W. Chou M.-Y. Iijima K. et al. (2020). Asian working group for sarcopenia: 2019 consensus update on sarcopenia diagnosis and treatment. *J. Am. Med. Dir. Assoc.* 21. 300–307.e2. 10.1016/j.jamda.2019.12.012 32033882

[B13] Chen Y. Al-Nusaif M. Li S. Tan X. Yang H. Cai H. et al. (2024). Progress on early diagnosing Alzheimer’s disease. *Front. Med*. 18:446–464. 10.1007/s11684-023-1047-1 38769282 PMC11391414

[B14] Chen Y. Zhan Y. Wang H. Zhang H. Cai Y. Wang L. et al. (2022). Mediating effect of lower extremity muscle strength on the relationship between mobility and cognitive function in Chinese older adults: a cross-sectional study. *Front. Aging Neurosci*. 14:984075. 10.3389/fnagi.2022.984075 36408099 PMC9669366

[B15] Ghahremani M. Smith E. E. Chen H.-Y. Creese B. Goodarzi Z. Ismail Z. (2023). Vitamin D supplementation and incident dementia: effects of sex, APOE, and baseline cognitive status. *Alzheimers Dement.* 15:e12404. 10.1002/dad2.12404 36874594 PMC9976297

[B16] Gonzalez-Gomez R. Demnitz N. Coronel C. Gates A. T. Kjaer M. Siebner H. R. et al. (2026). Randomized controlled trial of resistance exercise and brain aging clocks. *Geroscience* [Online ahead of print]. 10.1007/s11357-026-02141-x 41665740 PMC13356001

[B17] Isaka M. Sugimoto K. Yasunobe Y. Akasaka H. Fujimoto T. Kurinami H. et al. (2019). The usefulness of an alternative diagnostic method for sarcopenia using thickness and echo intensity of lower leg muscles in older males. *J. Am. Med. Dir. Assoc.* 20 1185.e1–1185.e8. 10.1016/j.jamda.2019.01.152 30902675

[B18] Jack C. R. Bennett D. A. Blennow K. Carrillo M. C. Dunn B. Haeberlein S. B. et al. (2018). NIA-AA research framework: toward a biological definition of Alzheimer’s disease. *J. Alzheimers Assoc.* 14 535–562. 10.1016/j.jalz.2018.02.018 29653606 PMC5958625

[B19] Jiang X. Guo Y. Cui L. Huang L. Guo Q. Huang G. (2023). Study of diet habits and cognitive function in the chinese middle-aged and elderly population: the association between folic acid, B Vitamins, Vitamin D, Coenzyme Q10 supplementation and cognitive ability. *Nutrients* 15:1243. 10.3390/nu15051243 36904242 PMC10005055

[B20] Jones E. J. Bishop P. A. Woods A. K. Green J. M. (2008). Cross-sectional area and muscular strength: a brief review. *Sports Med. Auckl. NZ.* 38 987–994. 10.2165/00007256-200838120-00003 19026016

[B21] Kang J. Park M. Lee E. Jung J. Kim T. (2022). The role of vitamin D in Alzheimer’s Disease: a transcriptional regulator of amyloidopathy and gliopathy. *Biomedicines* 10:1824. 10.3390/biomedicines10081824 36009371 PMC9404847

[B22] Kim J. Suh S. I. Park Y. J. Kang M. Chung S. J. Lee E. S. et al. (2024). Sarcopenia is a predictor for Alzheimer’s continuum and related clinical outcomes. *Sci. Rep*. 14:21074. 10.1038/s41598-024-62918-y 39256402 PMC11387779

[B23] Lee J. H. Kim Y. A. Kim Y. S. Lee Y. Seo J. H. (2023). Association between vitamin D deficiency and clinical parameters in men and women aged 50 years or older: a cross-sectional cohort study. *Nutrients* 15:3043. 10.3390/nu15133043 37447368 PMC10346261

[B24] Liu R. Guo Z. Li M. Liu S. Zhi Y. Jiang Z. et al. (2024). Lower fractional dimension in Alzheimer’s disease correlates with reduced locus coeruleus signal intensity. *Magn. Reson. Imaging* 106 24–30. 10.1016/j.mri.2023.08.001 37541457

[B25] Liu S. Zhang Y. Peng B. Pang C. Li M. Zhu J. et al. (2022). Correlation between parameters related to sarcopenia and gray matter volume in patients with mild to moderate Alzheimer’s disease. *Aging Clin. Exp. Res.* 34 3041–3053. 10.1007/s40520-022-02244-3 36121640

[B26] Liu S.-W. Ma X.-T. Yu S. Weng X.-F. Li M. Zhu J. et al. (2024). Bridging reduced grip strength and altered executive function: specific brain white matter structural changes in patients with Alzheimer’s Disease. *Clin. Interv. Aging* 19 93–107. 10.2147/CIA.S438782 38250174 PMC10799618

[B27] Lu L. Mao L. Yang S. He X. Zhang Z. Chen N. (2023). Gender differences in the association between sarcopenia and depressive symptoms among community-dwelling older people in a Chinese Suburban area. *J. Multidiscip. Healthc*. 16 3813–3824. 10.2147/JMDH.S439785 38076588 PMC10706056

[B28] Ma X. T. Liu S. W. Yap L. L. Bao R. Y. Li Y. F. Yang Y. F. et al. (2025). Research progress on the mechanism of inflammatory aging in the disease process of Alzheimer’s disease. *Pract. Geriatr.* 39 202–206. 10.3969/j.issn.1003-9198.2025.02.021

[B29] McCormick R. Vasilaki A. (2018). Age-related changes in skeletal muscle: changes to life-style as a therapy. *Biogerontology* 19 519–536. 10.1007/s10522-018-9775-3 30259289 PMC6223729

[B30] McKhann G. M. Knopman D. S. Chertkow H. Hyman B. T. Jack C. R. Kawas C. H. et al. (2011). The diagnosis of dementia due to Alzheimer’s disease: recommendations from the National Institute on Aging-Alzheimer’s Association workgroups on diagnostic guidelines for Alzheimer’s disease. *Alzheimers Dement*. 7 263–269. 10.1016/j.jalz.2011.03.005 21514250 PMC3312024

[B31] Moradi K. Albert M. Demehri S. (2024). *Skeletal Muscle Loss is Associated with Increased Risk of Dementia-Related Outcomes: Longitudinal Observational Study Using ADNI Brain MRIs.* Chicago, IL: Radiological Society of North America, Inc.

[B32] Navale S. S. Mulugeta A. Zhou A. Llewellyn D. J. Hyppönen E. (2022). Vitamin D and brain health: an observational and Mendelian randomization study. *Am. J. Clin. Nutr*. 116 531–540. 10.1093/ajcn/nqac107 35451454 PMC9348994

[B33] Nichols E. Szoeke C. Vollset S. Abbasi N. Abd-Allah F. Abdela J. et al. (2019). Global, regional, and national burden of Alzheimer’s disease and other dementias, 1990–2016: a systematic analysis for the Global Burden of Disease Study 2016. *Lancet Neurol.* 18, 88–106. 10.1016/S1474-4422(18)30403-4 30497964 PMC6291454

[B34] Ogawa Y. Kaneko Y. Sato T. Shimizu S. Kanetaka H. Hanyu H. (2018). Sarcopenia and muscle functions at various stages of Alzheimer Disease. *Front. Neurol*. 9:710. 10.3389/fneur.2018.00710 30210435 PMC6121095

[B35] Oudbier S. J. Goh J. Looijaard S. M. L. M. Reijnierse E. M. Meskers C. G. M. Maier A. B. (2022). Pathophysiological mechanisms explaining the association between low skeletal muscle mass and cognitive function. *J. Gerontol. A Biol. Sci. Med. Sci*. 77 1959–1968. 10.1093/gerona/glac121 35661882 PMC9536455

[B36] Oveisgharan S. Wang T. Barnes L. L. Schneider J. A. Bennett D. A. Buchman A. S. (2024). The time course of motor and cognitive decline in older adults and their associations with brain pathologies: a multicohort study. *Lancet Healthy Longev*. 5 e336–e345. 10.1016/S2666-7568(24)00033-3 38582095 PMC11129202

[B37] Pál É Ungvári Z. Benyó Z. Várbíró S. (2023). Role of vitamin D deficiency in the pathogenesis of cardiovascular and cerebrovascular diseases. *Nutrients* 15:334. 10.3390/nu15020334 36678205 PMC9864832

[B38] Pedersen B. K. (2019). Physical activity and muscle-brain crosstalk. *Nat. Rev. Endocrinol*. 15 383–392. 10.1038/s41574-019-0174-x 30837717

[B39] Peng T. C. Chen W. L. Wu L. W. Chang Y. W. Kao T. W. (2020). Sarcopenia and cognitive impairment: a systematic review and meta-analysis. *Clin. Nutr*. 39 2695–2701. 10.1016/j.clnu.2019.12.014 31917049

[B40] She J. M. Che J. Chen C. Y. Lu L. Y. Zhao Y. Yu R. X. et al. (2021). Advances in research on ultraviolet-induced skin photoaging and photocarcinogenesis. *Chin. J. Plast. Surg.* 37 220–224. 10.3760/cma.j.cn114453-20200608-00343 30704229

[B41] Shea M. K. Barger K. Dawson-Hughes B. Leurgans S. E. Fu X. James B. D. et al. (2023). Brain vitamin D forms, cognitive decline and neuropathology in community-dwelling older adults. *J. Alzheimers Assoc.* 19 2389–2396. 10.1002/alz.12836 PMC10244481 36479814

[B42] Shigemori K. Ohgi S. Okuyama E. Shimura T. Schneider E. (2010). The factorial structure of the Mini-Mental State Examination (MMSE) in Japanese dementia patients. *BMC Geriatr*. 10:36. 10.1186/1471-2318-10-36 20534132 PMC2903593

[B43] Su C. Zhang S. Zheng Q. Miao J. Guo J. (2025). Prevalence and correlation of sarcopenia with Alzheimer’s disease: a systematic review and meta-analysis. *PLoS One* 20:e0318920. 10.1371/journal.pone.0318920 40029915 PMC11875368

[B44] Ülger Z. Ayçiçek G. Ş Kara Ö Kara M. (2022). Ultrasonographic/regional muscle measurements for diagnosing sarcopenia in older adults with and without dementia. *Turk. J. Med. Sci.* 52 1926–1932. 10.55730/1300-0144.5540 36945995 PMC10390111

[B45] Van Hooren B. Aagaard P. Monte A. Blazevich A. J. (2024). The role of pennation angle and architectural gearing to rate of force development in dynamic and isometric muscle contractions. *Scand. J. Med. Sci. Sports* 34:e14639. 10.1111/sms.14639 38686976

[B46] van Schoor N. Lips P. (2017). Global overview of vitamin D status. *Endocrinol. Metab. Clin. North Am*. 46 845–870. 10.1016/j.ecl.2017.07.002 29080639

[B47] Varan H. D. Ceker E. Cataltepe E. Gungor F. Fadiloglu A. Borazan F. Y. (2024). Predictive value of adductor pollicis muscle thickness for ultrasound-based sarcopenia in older adults. *Nutr. Clin. Pract*. 39 619–625. 10.1002/ncp.11149 38699806

[B48] Weng X. Liu S. Li M. Zhang Y. Zhu J. Liu C. et al. (2023). White matter hyperintensities: a possible link between sarcopenia and cognitive impairment in patients with mild to moderate Alzheimer’s disease. *Eur. Geriatr. Med*. 14 1037–1047. 10.1007/s41999-023-00818-6 37330930

[B49] Ye C. Kong L. Wang Y. Zheng J. Xu M. Xu Y. et al. (2023). Causal associations of sarcopenia-related traits with cardiometabolic disease and Alzheimer’s disease and the mediating role of insulin resistance: a Mendelian randomization study. *Aging Cell* 22:e13923. 10.1111/acel.13923 37403750 PMC10497819

[B50] Zordoky B. N. El-Kadi A. O. (2009). Role of NF-kappaB in the regulation of cytochrome P450 enzymes. *Curr. Drug Metab*. 10 164–178. 10.2174/138920009787522151 19275551

