# Supplementary material for: From muscle to brain: the mediating effect of vitamin D in the relationship between muscle loss and cognitive function in Alzheimer’s disease
Source: Front Aging Neurosci. 2026 May 13;18:1630798. doi: 10.3389/fnagi.2026.1630798 (PMC13212239; doi:10.3389/fnagi.2026.1630798)
Supplement: Supplementary file 1 [file Data_Sheet_1.docx]

Supplementary materials for Section 2

**1. Testing Procedures and Settings**:

All these examinations were conducted in specialized environments to ensure data quality and standardization. Each participant underwent assessments into one day to ensure data quality. To reduce the effect of fatigue, rest intervals (approximately 5 minutes) were provided between different test modules.

General information was collected via structured interview and neuropsychological scales were collected via standardized assessment in a quiet room within the Memory Disorder Clinic by professional physicians.

Physical assessments were administered by professional physicians within the clinical examination room.

muscle ultrasound examinations were performed by specialized sonographers, who were blinded to the clinical status of the participants during data acquisition and analysis.

Neuroimaging examinations, including MRI and PET-CT, were scheduled through the standard outpatient clinical workflow and were conducted and interpreted by professional radiologists and nuclear medicine physicians. AD patients underwent both MRI and PET scans. Neurotypical controls only underwent MRI.

**2. Function of neuropsychological tests**

| **Scale Name** | **Assessment content** |
| --- | --- |
| **Non-cognitive assessments** |  |
| Hamilton depression scale (HAMD) | the severity of depressive symptoms |
| Hamilton anxiety scale (HAMA) | the severity of anxiety symptoms, including both somatic and psychic anxiety. |
| Pittsburgh sleep quality index (PSQI) | quality of sleepiness |
| Athens insomnia scale (AIS) | the severity of insomnia based on the ICD-10 diagnostic criteria for insomnia |
| Epworth sleepiness scale (ESS) | the severity of daytime sleepiness |
| **Cognitive function assessments** |  |
| the Chinese version of the MMSE | comprehensive cognitive functioning |
| the Beijing version of the Montreal cognitive assessment (MoCA) | comprehensive cognitive functioning |
| the memory and executive screening scale (MES) | MES-T includes the Memory Factor Score (MES-M), which assesses episodic memory, and the Executive Factor Score (MES-E) to assess executive function |
| the clock drawing test (CDT) | executive and visuospatial abilities |
| animal fluency task (AFT) | executive and language abilities |
| Boston naming test (BNT) | naming abilities |
| digital span test (DST) | DST contains both forward (FDST) and backward (BDST) components to assess attention, with the BDST also assessing working memory for executive function |
| digit symbol substitution test (DSST) | speed of information processing, which considered kind of executive function. |
| clinical dementia rating (CDR): CDR-global score (CDR-GS). | severity of dementia |

Table S1. Details of neuropsychological tests

**3. Detailed testing procedures of muscle ultrasound**

The examiner's right forearm was placed on a custom-made handrest, and the probe lightly touched the skin surface to increase the stability of the right hand and reduce the pressure of the forearm and the probe on the muscles. Firstly, the muscles of arm were measured, with the subject lying supine, both elbows fully extended and placed by the sides of the body with palms facing upwards, allowing relaxation of the brachioradialis and Biceps brachii. Two-dimensional grayscale ultrasound was applied, with the probe placed along the longitudinal axis of the muscle belly at the thickest areas of the brachioradialis and biceps. Relevant organic lesions such as muscle tears, hematomas, and edema were excluded, and the probe was positioned away from fascia, bone, and large blood vessels. Once the image stabilized, MT and CSA were measured. Next, switched to SWE mode, and set the sampling box to (8-10) mm × (8-10) mm in the region of interest at a depth of 1.2 cm. After the image stabilizes, select the region with uniform colors (red indicating high stiffness, blue indicating low stiffness), and activate the quantitative analysis system (Q-BOX) to automatically calculate the average YM in a 4 mm diameter circular ROI.

Secondarily, the measurement of lower limb muscles is performed with the subject in a prone position, with both feet naturally hanging off the edge of the examination table and the legs extended to achieve a neutral ankle position, indicating the relaxed state of the gastrocnemius. In this state, the gastrocnemius may be slightly stretched due to gravity, but there is no active contraction, and it relies on soft tissue elasticity to maintain its form. Keeping the prone position, the ankle joint is maximally plantar-flexed, placing the gastrocnemius in a contracted state, where it actively contracts. When the subject stands with their feet shoulder-width apart, this is considered the tense state of the gastrocnemius, where the muscle undergoes isometric contraction, meaning the internal muscle fibers generate continuous force without altering the muscle's overall length. The gastrocnemius helps stabilize the ankle joint, maintain balance, and counteract gravity to sustain an upright posture^23^ . In the longitudinal section the probe is placed lightly on the medial head of the gastrocnemius, parallel to the long axis of the muscle. The ROI grey scale image was first displayed and MT, CSA, FL, PA were measured in a relaxed state, then switched to SWE mode to obtain the YM mean. The same method was used to measure FL, PA, and YM averages in the contracted and tense states.
